# Supplementary material for: Association between eczema and major cardiovascular outcomes in population-based studies: a systematic review protocol
Source: BMJ Open. 2017 Sep 29;7(9):e017979. doi: 10.1136/bmjopen-2017-017979 (PMC5640133; doi:10.1136/bmjopen-2017-017979)
Supplement: Supplementary file 1 [file bmjopen-2017-017979supp001.pdf]

Supplementary Appendix 1

Ovid MEDLINE Search Strategy

1. Dermatitis, Atopic/

2. exp Eczema/

3. (eczem* or atopic dermatit*).mp. [mp=title, abstract, original title, name of substance word, subject heading word, keyword heading word, protocol supplementary concept word, rare disease supplementary concept word, unique identifier, synonyms]

4. or/1-3

5. intracranial embolism/

6. exp Intracranial Hemorrhages/

7. exp Intracranial Arterial Diseases/

8. exp Brain Ischemia/

9. (intracranial embolism* or intracranial h?emorrhage* or intracranial arterial disease* or intracranial thrombos* or stroke* or cerebrovascular accident* or cerebrovascular diseas* or cva or cerebral artery diseas* or brain isch* or brain infarct* or brain h?emorrhag* or occlusive cerebrovascular disease*).mp. [mp=title, abstract, original title, name of substance word, subject heading word, keyword heading word, protocol supplementary concept word, rare disease supplementary concept word, unique identifier, synonyms]

10. exp Cardiovascular Diseases/

11. (myocardial isch?emia* or heart arrest* or heart attack* or myocardial infarct* or acute coronary syndrome* or angina* or isch?emic heart diseas* or coronary arter* or heart fail* or congestive cardiac fail* or ccf or lvf or left ventricular fail* or rvf or right ventricular fail* or heart right ventricle fail* or heart left ventricle fail* or af or cardiovascular diseas* or heart diseas* or vascular diseas* or arrhythmia* or abnormal heart rhythm* or atrial flutter* or heart block* or svt* or supraventricular tachycardia* or bundle branch block*).mp. [mp=title, abstract, original title, name of substance word, subject heading word, keyword heading word, protocol supplementary concept word, rare disease supplementary concept word, unique identifier, synonyms]

12. exp Myocardial Revascularization/

13. exp Percutaneous Coronary Intervention/

14. (myocardial revasculari?ation* or percutaneous coronary intervention* or heart muscle revasculari?ation* or coronary revasculari?ation* or percutaneous transluminal angioplast* or coronary artery obstruction* or transluminal coronary angioplast* or coronary artery surger* or interventional cardiovascular procedure*).mp. [mp=title, abstract, original title, name of substance word, subject heading word, keyword heading word, protocol supplementary concept word, rare disease supplementary concept word, unique identifier, synonyms]

15. (cardiac arrest* or heart death* or card* death* or (cardiovascular adj3 mortalit*)).mp. [mp=title, abstract, original title, name of substance word, subject heading word, keyword heading word, protocol supplementary concept word, rare disease supplementary concept word, unique identifier, synonyms]

16. or/5-15

17. 4 and 16
