# Supplementary material for: Association between eczema and major cardiovascular outcomes in population-based studies: a systematic review protocol
Source: BMJ Open. 2017 Sep 29;7(9):e017979. doi: 10.1136/bmjopen-2017-017979 (PMC5640133; doi:10.1136/bmjopen-2017-017979)
Supplement: Author's manuscript [file bmjopen-2017-017979.draft_revisions.pdf]

BMJ Open is committed to open peer review. As part of this commitment we make the peer review history of every article we publish publicly available.

When an article is published we post the peer reviewers' comments and the authors' responses online. We also post the versions of the paper that were used during peer review. These are the versions that the peer review comments apply to.

The versions of the paper that follow are the versions that were submitted during the peer review process. They are not the versions of record or the final published versions. They should not be cited or distributed as the published version of this manuscript.

BMJ Open is an open access journal and the full, final, typeset and author-corrected version of record of the manuscript is available on our site with no access controls, subscription charges or pay-per-view fees (http://bmjopen.bmj.com).

If you have any questions on BMJ Open's open peer review process please email info.bmjopen@bmj.com

The association between eczema and major cardiovascular outcomes in population based studies: a systematic review protocol

Journal:	*BMJ Open*
Manuscript ID	bmjopen-2017-017979
Article Type:	Protocol
Date Submitted by the Author:	30-May-2017
Complete List of Authors:	Ascott, Anna; London School of Hygiene and Tropical Medicine,<br>Yu, Ashley; University of Ottawa Faculty of Medicine<br>Schmidt, Morten; Aarhus University, Department of Clinical Epidemiology<br>Abuabara, Katrina; University of California<br>Smeeth, Liam; London School of Hygiene and Tropical Medicine, Epidemiology and Population Health<br>Langan, Sinead; London School of Hygiene and Tropical Medicine, Epidemiology and Population Health
**Primary Subject Heading**:	Epidemiology
Secondary Subject Heading:	Dermatology, Cardiovascular medicine
Keywords:	EPIDEMIOLOGY, Dermatological epidemiology < DERMATOLOGY, Cardiac Epidemiology < CARDIOLOGY, Eczema < DERMATOLOGY, DERMATOLOGY, CARDIOLOGY

The association between eczema and major cardiovascular outcomes in population based studies: a systematic review protocol

Anna Ascott[1], Ashley M Yu[2], Morten Schmidt[3], Katrina Abuabara[4], Liam Smeeth[1], Sinéad M Langan[1]

Corresponding author: Anna Ascott. Email: annaascott@doctors.org.uk. Tel: 07837517279.

Author affiliations:

[1]Faculty of Epidemiology & Population Health, London School of Hygiene and Tropical Medicine, UK

[2]Faculty of Medicine, University of Ottawa, Canada

[3]Department of Clinical Epidemiology, Aarhus University Hospital, Denmark

[4]School of Medicine, University of California, USA

Word count: 1408

Table and figure count: 0

For peer review only

ABSTRACT

Introduction: Chronic inflammatory diseases such as eczema (also known as atopic dermatitis) have been inconsistently linked to cardiovascular disease and stroke in both mechanistic and epidemiological studies. There is a need to review the existing epidemiological data examining the association between eczema and major cardiovascular outcomes including angina, myocardial infarction, coronary revascularisation, heart failure, cardiac arrhythmias, stroke, and cardiovascular death, in order to improve our understanding of the comorbidities of eczema.

Methods and analysis: We will systematically review population based studies, including cohort, case-control, and cross-sectional studies, reporting on the association between eczema and cardiovascular outcomes. We will search MEDLINE, EMBASE, and Global Health, from their date of inception to April 2017, using a comprehensive search strategy formulated with the help of a librarian. Two reviewers will independently screen titles and abstracts in duplicate, followed by independent data extraction and quality assessment. We will group studies by the cardiovascular outcome under study, and synthesise them narratively. If sufficient numbers of homogenous studies are returned, we will perform meta-analyses to obtain a pooled effect estimates. Preferred Reporting Items for Systematic Review and Meta-Analysis (PRISMA) will be used to inform the reporting of this study.

Ethics and dissemination: Ethical approval is not required as this is a systematic review. The results of this review will be submitted for peer-reviewed publication, and for national and international presentation.

PROSPERO registration number: CRD42017060359

For peer review only

STRENGTHS AND LIMITATIONS OF THIS STUDY

Strengths:

- This systematic review protocol is reported in line with Preferred Reporting Items for Systematic Review and Meta-Analysis Protocols (PRISMA-P) guidelines[1].
- A systematic and broad search strategy planned with the help of a librarian attempts to identify all studies that meet the eligibility criteria.
- Two authors will independently assess search results using an online data management programme to minimise bias and errors in screening, data extraction and quality assessment of studies.

Limitations:

- Despite an extensive search, it is still possible that relevant papers may have been missed.
- Studies may not systematically report all outcomes.

For peer review only

INTRODUCTION

Eczema is an inflammatory skin disease, traditionally considered a disease of childhood. However, eczema may affect around 10% of adults[2 3] and the global prevalence of eczema is increasing[4]. Concurrently, cardiovascular disease is fast becoming a significant cause of mortality and morbidity in both high and low and middle income countries. Chronic inflammatory conditions have been linked to cardiovascular disease, in diseases of varying aetiology, from psoriasis and rheumatoid arthritis to HIV[5-8].

There are a number of lines of evidence supporting an association between eczema and cardiovascular disease. Mechanistic studies suggest that platelet dysfunction and decreased fibrinolysis may contribute to increased clotting in eczema[9 10]. Severe eczema has been associated with increased incidence of coronary artery disease using cardiac computed tomography angiography (CCTA)[11]. In addition, treatments used for eczema may increase cardiovascular risk[12 13], however, a likely limitation of the studies to be included in this review will be the likely strong collinearity between eczema severity and level of treatment. Factors such as confounding by severity could contribute to an observed increase in cardiovascular disease and stroke in eczema.

Epidemiological studies have inconsistently linked eczema to cardiovascular risk factors, disease, and stroke across different populations[14-17], whilst previous systematic reviews have found an association between eczema and risk factors for cardiovascular disease including raised BMI[18] and childhood type 1 diabetes[19]. There is a need to review the existing epidemiological data linking eczema to major cardiovascular outcomes in order to increase our understanding of the comorbidities of eczema, and to inform management and prevention strategies at an individual and population level.

The primary objective of this systematic review is to ascertain the association between eczema and major cardiovascular outcomes including angina, myocardial infarction, coronary revascularisation, heart failure, cardiac arrhythmias, stroke, and cardiovascular death, in population based studies.

For peer review only

The secondary objectives of this review are to answer the following questions:

- Does the strength of the association between eczema and major cardiovascular outcomes increase as severity of eczema increases?
- Does treatment of eczema alter the risk of cardiovascular outcomes (although it may not be possible to completely disentangle the effects of disease severity and treatment as those with severe disease are often defined by their treatment with systemic therapy)?
- Is the association between eczema and major cardiovascular outcomes consistent across different countries?
- Does the association vary for different CVD endpoints?
- Is there any modification of effect by age and gender?

METHODS AND ANALYSIS

Eligibility criteria

Peer reviewed, published studies in any language, from any year, are eligible to be included. Studies must be population based, with average age of participants >18. Studies may be cohort, case-control, or cross-sectional designs, from any healthcare setting, with any length of follow up. The exposure of interest is eczema (atopic dermatitis). The comparator will be people or person years without eczema. Outcomes are major cardiovascular outcomes, which will include stroke, myocardial infarction, heart failure, cardiac arrhythmias, angina, coronary revascularisation and cardiovascular death.

We will exclude case series (including retrospective clinic populations), ecological studies, reviews, and studies in paediatric populations only. Studies of localised or other types of eczema such a hand eczema, and seborrheic or contact dermatitis, are not eligible to be included.

Literature search

For peer review only

We will search the following electronic databases from their date of inception to April 2017: MEDLINE via Ovid, EMBASE via Ovid, and Global Health. The search strategies were created by a researcher with medical and systematic review training, in conjunction with a librarian with expertise in searching the literature, and reviewed by all authors. Exclusion filters and limits will not be used in the search due to the risk that eligible studies may be inadvertently excluded. The Ovid MEDLINE search strategy is available to view (see online supplementary appendix 1). We will also review the bibliographies of included studies, and contact experts in the field for relevant references.

Selection of studies and data extraction

Covidence, an online literature reviewing data management programme, will be used to facilitate collaboration and data extraction between reviewers. All titles and abstracts resulting from the literature search will be uploaded to Covidence. Duplicates will be removed by AA. Two reviewers (AA and AY) will independently screen titles and abstracts in duplicate. Full text articles will be retrieved where studies fulfil inclusion criteria, or where there is any ambiguity of the study's eligibility. Disagreement will be resolved through discussion with a third reviewer where necessary. Additional information will be requested from authors if needed. Review authors will not be blinded to the journal titles or study authors. A PRISMA flow diagram[20] will document the process of literature selection, and reasons for exclusion.

Each reviewer will extract data independently and in duplicate in order to minimise bias and errors, using a standardised data extraction tool, which will be piloted on the first three eligible texts to ensure its suitability. Data will be sought for the following domains:

1. Study details: author information, publication year, design, sponsorship, geographical location, healthcare setting, length of follow up time if relevant, sampling and recruitment methods, period of study, aims and objectives;
2. Population characteristics: e.g. mean and median age, inclusion and exclusion criteria;

For peer review only

3. Exposure: definition of eczema as an exposure, number of exposed subjects, details of eczema severity and treatment, age at onset of eczema;
4. Comparators: definition of unexposed subjects, number of comparators;
5. Outcomes: definition and identification of cardiovascular outcomes (stroke, myocardial infarction, heart failure, cardiac arrhythmias, angina, coronary revascularisation and cardiovascular death), number of subjects with the outcome;

Where available, unadjusted and fully adjusted effect estimates will be recorded, along with details of confounders. Discrepancies will be resolved by discussion, with a third reviewer if necessary.

Outcomes

The primary outcome is the association between eczema and cardiovascular outcomes including stroke (all subtypes), myocardial infarction, heart failure, cardiac arrhythmias, angina, coronary revascularisation and cardiovascular death. Where possible, effect estimates will include odds ratios, hazard ratios, and incident rate ratios, for cardiovascular outcomes in people with eczema compared to those without. Secondary outcomes include variation in the strength of association between severity of eczema and cardiovascular disease, whether the association varies by country, is altered by the treatment of eczema, by different CVD endpoints, or modified by age and gender.

Quality assessment

Critical appraisal will be independently recorded by reviewers to allow comparison by study quality. Risk of bias will be assessed by considering relevant domains to observational studies, including participant selection, measurement of variables, and controlling for confounding, in line with the Cochrane Collaboration's tool for assessing risk of bias, and the Newcastle-Ottawa Scale, in order to maximise relevance to non-randomised studies. Each domain will be rated with ‘high’, ‘low’ or ‘unclear’ with regards to the risk of bias, with free text explanations. Full results of this quality

For peer review only

assessment will be included in the resulting review, with discussion of quality assessment in the narrative data synthesis.

Data synthesis

We will group studies by the cardiovascular outcome under study, and synthesise them narratively. No subgroup analyses are planned. If sufficient numbers of homogenous studies are returned, we will perform a meta-analysis with the help of a statistician to obtain a pooled effect estimate. Study characteristics and the effect estimates for the association between eczema and cardiovascular disease will be presented in full, in tabular form. Studies at high risk of bias will not be contained in the synthesis. Preferred Reporting Items for Systematic Review and Meta-Analysis (PRISMA) guidelines[20] will be used to report the results of this study.

ETHICS AND DISSEMINATION

This systematic review protocol was registered with the International Prospective Register of Systematic Reviews (PROSPERO) on the 22nd of April 2016. Any amendments to the protocol will be documented on the PROSPERO site contemporaneously, with full explanation of any change.

Ethical approval is not required for this study as it is a systematic review. The results will be submitted for peer-reviewed publication, and for national and international presentation.

Authors' contributions:

AA contributed to the design of the study, was the guarantor, developed the search strategy and the PROSPERO protocol, and drafted the manuscript. AY contributed to the design of the study, approved the search strategy, and provided critical feedback on the PROSPERO protocol, and the final manuscript. MS approved the search strategy, and provided critical feedback on the PROSPERO protocol and the final manuscript. KA approved the search strategy, and provided critical feedback on the PROSPERO protocol and the final manuscript. LS approved the search strategy, and provided critical feedback on the PROSPERO protocol and the final manuscript. SML contributed to the

For peer review only

conception and design of the study, and provided critical feedback on the search strategy, PROSPERO protocol and the final manuscript.

Funding statement: Publication of this manuscript was funded by a Wellcome Senior Clinical Fellowship to SML (205039/Z/16/Z). AA was supported by a small grant from the British Association of Dermatologists. LS is funded by a Wellcome Trust senior fellowship in clinical science. The Wellcome Trust and the British Association of Dermatologists played no role in the development of this study or the protocol.

Competing interests statement: All authors have completed the ICMJE uniform disclosure form at www.icmje.org/coi_disclosure.pdf and declare: AA reports grants from the British Association of Dermatologists during the conduct of the study. LS reports grants from Wellcome Trust during the conduct of the study; grants from Wellcome Trust, Medical Research Council, National Institute for Health Research and the European Union outside the submitted work, personal fees from GSK for advisory work unrelated to the submitted work, grant funding from GSK for academic research unrelated to the submitted work, acts as an unpaid steering committee chair for AstraZeneca for a randomised trial unrelated to the submitted work, and is also a trustee of the British Heart Foundation. SML reports grants from Wellcome Senior Clinical fellowship in Science (205039/Z/16/Z) during the conduct of the study.

REFERENCES

1. Moher D, Shamseer L, Clarke M, et al. Preferred reporting items for systematic review and meta-analysis protocols (PRISMA-P) 2015 statement. *Syst Rev* 2015;4:1. doi: 10.1186/2046-4053-4-1
2. Ronmark EP, Ekerljung L, Lotvall J, et al. Eczema among adults: prevalence, risk factors and relation to airway diseases. Results from a large-scale population survey in Sweden. *Br J Dermatol* 2012;166(6):1301-8. doi: 10.1111/j.1365-2133.2012.10904.x
3. Silverberg JI, Hanifin JM. Adult eczema prevalence and associations with asthma and other health and demographic factors: a US population-based study. *J Allergy Clin Immunol* 2013;132(5):1132-8. doi: 10.1016/j.jaci.2013.08.031
4. Williams H, Stewart A, von Mutius E, et al. Is eczema really on the increase worldwide? *J Allergy Clin Immunol* 2008;121(4):947-54 e15. doi: 10.1016/j.jaci.2007.11.004
5. Hansson GK. Inflammation, atherosclerosis, and coronary artery disease. *N Engl J Med* 2005;352(16):1685-95. doi: 10.1056/NEJMra043430

For peer review only

6. Hsue PY, Deeks SG, Hunt PW. Immunologic basis of cardiovascular disease in HIV-infected adults. *J Infect Dis* 2012;205 Suppl 3:S375-82. doi: 10.1093/infdis/jis200
7. Roifman I, Beck PL, Anderson TJ, et al. Chronic inflammatory diseases and cardiovascular risk: a systematic review. *Can J Cardiol* 2011;27(2):174-82. doi: 10.1016/j.cjca.2010.12.040
8. Mehta NN, Azfar RS, Shin DB, et al. Patients with severe psoriasis are at increased risk of cardiovascular mortality: cohort study using the General Practice Research Database. *Eur Heart J* 2010;31(8):1000-06. doi: 10.1093/eurheartj/ehp567
9. Tamagawa-Mineoka R, Katoh N, Ueda E, et al. Platelet-derived microparticles and soluble P-selectin as platelet activation markers in patients with atopic dermatitis. *Clin Immunol* 2009;131(3):495-500. doi: 10.1016/j.clim.2009.01.006
10. Tamagawa-Mineoka R, Katoh N, Ueda E, et al. Elevated platelet activation in patients with atopic dermatitis and psoriasis: increased plasma levels of beta-thromboglobulin and platelet factor 4. *Allergol Int* 2008;57(4):391-6. doi: 10.2332/allergolint.O-08-537
11. Hjuler KF, Bottcher M, Vestergaard C, et al. Increased Prevalence of Coronary Artery Disease in Severe Psoriasis and Severe Atopic Dermatitis. *Am J Med* 2015;128(12):1325-34 e2. doi: 10.1016/j.amjmed.2015.05.041
12. Roubille C, Richer V, Starnino T, et al. The effects of tumour necrosis factor inhibitors, methotrexate, non-steroidal anti-inflammatory drugs and corticosteroids on cardiovascular events in rheumatoid arthritis, psoriasis and psoriatic arthritis: a systematic review and meta-analysis. *Ann Rheum Dis* 2015;74(3):480-9. doi: 10.1136/annrheumdis-2014-206624
13. Wei L, MacDonald TM, Walker BR. Taking glucocorticoids by prescription is associated with subsequent cardiovascular disease. *Ann Intern Med* 2004;141(10):764-70.
14. Standl M, Tesch F, Baurecht H, et al. Association of Atopic Dermatitis with Cardiovascular Risk Factors and Diseases. *J Invest Dermatol* 2017;137(5):1074-81. doi: 10.1016/j.jid.2016.11.031
15. Su VY, Chen TJ, Yeh CM, et al. Atopic dermatitis and risk of ischemic stroke: a nationwide population-based study. *Ann Med* 2014;46(2):84-9. doi: 10.3109/07853890.2013.870018
16. Riis JL, Vestergaard C, Hjuler KF, et al. Hospital-diagnosed atopic dermatitis and long-term risk of myocardial infarction: a population-based follow-up study. *BMJ Open* 2016;6(11):e011870. doi: 10.1136/bmjopen-2016-011870
17. Silverberg JI, Greenland P. Eczema and cardiovascular risk factors in 2 US adult population studies. *J Allergy Clin Immunol* 2015;135(3):721-8 e6. doi: 10.1016/j.jaci.2014.11.023
18. Zhang A, Silverberg JI. Association of atopic dermatitis with being overweight and obese: a systematic review and metaanalysis. *J Am Acad Dermatol* 2015;72(4):606-16 e4. doi: 10.1016/j.jaad.2014.12.013
19. Cardwell CR, Shields MD, Carson DJ, et al. A meta-analysis of the association between childhood type 1 diabetes and atopic disease. *Diabetes Care* 2003;26(9):2568-74.
20. Moher D, Liberati A, Tetzlaff J, et al. Preferred reporting items for systematic reviews and meta-analyses: the PRISMA statement. *BMJ* 2009;339:b2535. doi: 10.1136/bmj.b2535

For peer review only

Supplementary Appendix 1

Ovid MEDLINE Search Strategy

1. Dermatitis, Atopic/

2. exp Eczema/

3. (eczem* or atopic dermatit*).mp. [mp=title, abstract, original title, name of substance word, subject heading word, keyword heading word, protocol supplementary concept word, rare disease supplementary concept word, unique identifier, synonyms]

4. or/1-3

5. intracranial embolism/

6. exp Intracranial Hemorrhages/

7. exp Intracranial Arterial Diseases/

8. exp Brain Ischemia/

9. (intracranial embolism* or intracranial h?emorrhage* or intracranial arterial disease* or intracranial thrombos* or stroke* or cerebrovascular accident* or cerebrovascular diseas* or cva or cerebral artery diseas* or brain isch* or brain infarct* or brain h?emorrhag* or occlusive cerebrovascular disease*).mp. [mp=title, abstract, original title, name of substance word, subject heading word, keyword heading word, protocol supplementary concept word, rare disease supplementary concept word, unique identifier, synonyms]

10. exp Cardiovascular Diseases/

11. (myocardial isch?emia* or heart arrest* or heart attack* or myocardial infarct* or acute coronary syndrome* or angina* or isch?emic heart diseas* or coronary arter* or heart fail* or congestive cardiac fail* or ccf or lvf or left ventricular fail* or rvf or right ventricular fail* or heart right ventricle fail* or heart left ventricle fail* or af or cardiovascular diseas* or heart diseas* or vascular diseas* or arrhythmia* or abnormal heart rhythm* or atrial flutter* or heart block* or svt* or supraventricular tachycardia* or bundle branch block*).mp. [mp=title, abstract, original title, name of substance word, subject heading word, keyword heading word, protocol supplementary concept word, rare disease supplementary concept word, unique identifier, synonyms]

12. exp Myocardial Revascularization/

13. exp Percutaneous Coronary Intervention/

14. (myocardial revasculari?ation* or percutaneous coronary intervention* or heart muscle revasculari?ation* or coronary revasculari?ation* or percutaneous transluminal angioplast* or coronary artery obstruction* or transluminal coronary angioplast* or coronary artery surger* or interventional cardiovascular procedure*).mp. [mp=title, abstract, original title, name of substance word, subject heading word, keyword heading word, protocol supplementary concept word, rare disease supplementary concept word, unique identifier, synonyms]

15. (cardiac arrest* or heart death* or card* death* or (cardiovascular adj3 mortalit*)).mp. [mp=title, abstract, original title, name of substance word, subject heading word, keyword heading word, protocol supplementary concept word, rare disease supplementary concept word, unique identifier, synonyms]

16. or/5-15

17. 4 and 16

For peer review only

The association between eczema and major cardiovascular outcomes in population based studies: a systematic review protocol

Journal:	*BMJ Open*
Manuscript ID	bmjopen-2017-017979.R1
Article Type:	Protocol
Date Submitted by the Author:	22-Aug-2017

Complete List of Authors:	Ascott, Anna; London School of Hygiene and Tropical Medicine,<br>Yu, Ashley; University of Ottawa Faculty of Medicine<br>Schmidt, Morten; Aarhus University, Department of Clinical Epidemiology<br>Abuabara, Katrina; University of California<br>Smeeth, Liam; London School of Hygiene and Tropical Medicine, Epidemiology and Population Health<br>Langan, Sinead; London School of Hygiene and Tropical Medicine, Epidemiology and Population Health

**Primary Subject Heading**:	Epidemiology
Secondary Subject Heading:	Dermatology, Cardiovascular medicine
Keywords:	EPIDEMIOLOGY, Dermatological epidemiology < DERMATOLOGY, Cardiac Epidemiology < CARDIOLOGY, Eczema < DERMATOLOGY, DERMATOLOGY, CARDIOLOGY

SCHOLARONE™
Manuscripts

The association between eczema and major cardiovascular outcomes in population based studies: a systematic review protocol

Anna Ascott[1], Ashley M Yu[2], Morten Schmidt[3], Katrina Abuabara[4], Liam Smeeth[1], Sinéad M Langan[1]

Corresponding author: Anna Ascott. Email: annaascott@doctors.org.uk. Tel: 07837517279.

Author affiliations:

[1]Faculty of Epidemiology & Population Health, London School of Hygiene and Tropical Medicine, UK

[2]Faculty of Medicine, University of Ottawa, Canada

[3]Department of Clinical Epidemiology, Aarhus University Hospital, Denmark

[4]School of Medicine, University of California, USA

Word count: **2192**

Table and figure count: 0

For peer review only

For peer review only - http://bmjopen.bmj.com/site/about/guidelines.xhtml

ABSTRACT

Introduction: Chronic inflammatory diseases such as eczema (also known as atopic dermatitis) have been inconsistently linked to cardiovascular disease and stroke in both mechanistic and epidemiological studies. There is a need to review the existing epidemiological data examining the association between eczema and major cardiovascular outcomes including angina, myocardial infarction, coronary revascularisation, heart failure, cardiac arrhythmias, stroke, and cardiovascular death, in order to improve our understanding of the comorbidities of eczema.

Methods and analysis: We will systematically review population based studies, including cohort, case-control, and cross-sectional studies, reporting on the association between eczema and cardiovascular outcomes. We will search MEDLINE, EMBASE, and Global Health, from their date of inception to April 2017, using a comprehensive search strategy formulated with the help of a librarian. Two reviewers will independently screen titles and abstracts in duplicate, followed by independent data extraction and quality assessment. We will group studies by the cardiovascular outcome under study, and synthesise them narratively. If sufficient numbers of homogenous studies are returned, we will perform meta-analyses to obtain a pooled effect estimates. Preferred Reporting Items for Systematic Review and Meta-Analysis (PRISMA) will be used to inform the reporting of this study.

Ethics and dissemination: Ethical approval is not required as this is a systematic review. The results of this review will be submitted for peer-reviewed publication, and for national and international presentation.

PROSPERO registration number: CRD42017060359

For peer review only

For peer review only - http://bmjopen.bmj.com/site/about/guidelines.xhtml

STRENGTHS AND LIMITATIONS OF THIS STUDY

Strengths:

- This systematic review protocol is reported in line with Preferred Reporting Items for Systematic Review and Meta-Analysis Protocols (PRISMA-P) guidelines.
- A systematic and broad search strategy planned with the help of a librarian attempts to identify all studies that meet the eligibility criteria.
- Two authors will independently assess search results using an online data management programme to minimise bias and errors in screening, data extraction and quality assessment of studies.

Limitations:

- Despite an extensive search, it is still possible that relevant papers may have been missed.
- Studies may not systematically report all outcomes.

For peer review only

For peer review only - http://bmjopen.bmj.com/site/about/guidelines.xhtml

INTRODUCTION

Eczema is an inflammatory skin disease, traditionally considered a disease of childhood. However, eczema may affect around 10% of adults[1 2] and the global prevalence of eczema is increasing[3]. Concurrently, cardiovascular disease is fast becoming a significant cause of mortality and morbidity in both high and low and middle income countries. Chronic inflammatory conditions have been linked to cardiovascular disease, in diseases of varying aetiology, from psoriasis and rheumatoid arthritis to HIV[4-7].

There are a number of lines of evidence supporting an association between eczema and cardiovascular disease. Mechanistic studies suggest that platelet dysfunction and decreased fibrinolysis may contribute to increased clotting in eczema[8 9]. Severe eczema has been associated with increased incidence of coronary artery disease using cardiac computed tomography angiography (CCTA)[10]. In addition, treatments used for eczema may increase cardiovascular risk[11 12], however, a likely limitation of the studies to be included in this review will be the likely strong collinearity between eczema severity and level of treatment. Factors such as confounding by severity could contribute to an observed increase in cardiovascular disease and stroke in eczema.

Epidemiological studies have inconsistently linked eczema to cardiovascular risk factors, disease, and stroke across different populations[13-16], whilst previous systematic reviews have found an association between eczema and risk factors for cardiovascular disease including raised BMI[17] and childhood type 1 diabetes[18]. There is a need to review the existing epidemiological data linking eczema to major cardiovascular outcomes in order to increase our understanding of the comorbidities of eczema, and to inform management and prevention strategies at an individual and population level.

The primary objective of this systematic review is to ascertain the association between eczema and major cardiovascular outcomes including angina, myocardial infarction, coronary revascularisation, heart failure, cardiac arrhythmias, stroke, and cardiovascular death, in population based studies.

For peer review only - http://bmjopen.bmj.com/site/about/guidelines.xhtml

The secondary objectives of this review are to answer the following questions:

- Does the strength of the association between eczema and major cardiovascular outcomes increase as severity of eczema increases?
- Does treatment of eczema alter the risk of cardiovascular outcomes (although it may not be possible to completely disentangle the effects of disease severity and treatment as those with severe disease are often defined by their treatment with systemic therapy)?
- Is the association between eczema and major cardiovascular outcomes consistent across different countries?
- Does the association vary for different CVD endpoints?
- Is there any modification of effect by age and gender?

METHODS AND ANALYSIS

Eligibility criteria

Peer reviewed, published studies in any language, from any year, are eligible to be included. Studies must be population based, with average age of participants >18. Studies may be cohort, case-control, or cross-sectional designs, from any healthcare setting, with any length of follow up. The exposure of interest is eczema (atopic dermatitis). The comparator will be people or person years without eczema. Outcomes are major cardiovascular outcomes, which will include angina, myocardial infarction, coronary revascularisation, heart failure, cardiac arrhythmias, stroke, and cardiovascular death.

We will exclude case series (including retrospective clinic populations), ecological studies, reviews, and studies in paediatric populations only. Studies of localised or other types of eczema such a hand eczema, and seborrheic or contact dermatitis, are not eligible to be included.

Literature search

For peer review only

We will search the following electronic databases from their date of inception to April 2017: MEDLINE via Ovid, EMBASE via Ovid, and Global Health. The search strategies were created by a researcher with medical and systematic review training, in conjunction with a librarian with expertise in searching the literature, and reviewed by all authors. Exclusion filters and limits will not be used in the search due to the risk that eligible studies may be inadvertently excluded. The Ovid MEDLINE search strategy is available to view (see online supplementary appendix 1). We will also review the bibliographies of included studies, and contact experts in the field for relevant references.

Selection of studies and data extraction

Covidence, an online literature reviewing data management programme, will be used to facilitate collaboration and data extraction between reviewers. All titles and abstracts resulting from the literature search will be uploaded to Covidence. Duplicates will be removed by AA. Two reviewers (AA and AY) will independently screen titles and abstracts in duplicate. Full text articles will be retrieved where studies fulfil inclusion criteria, or where there is any ambiguity of the study's eligibility. Disagreement will be resolved through discussion with a third reviewer where necessary. Additional information will be requested from authors if needed. Review authors will not be blinded to the journal titles or study authors. A PRISMA flow diagram[19] will document the process of literature selection, and reasons for exclusion.

Each reviewer will extract data independently and in duplicate in order to minimise bias and errors, using a standardised data extraction tool, which will be piloted on the first three eligible texts to ensure its suitability. Data will be sought for the following domains:

1. Study details: author information, publication year, design, sponsorship, geographical location, healthcare setting, length of follow up time if relevant, sampling and recruitment methods, period of study, aims and objectives;
2. Population characteristics: e.g. mean and median age, inclusion and exclusion criteria;

For peer review only - http://bmjopen.bmj.com/site/about/guidelines.xhtml

3. Exposure: definition of eczema as an exposure, number of exposed subjects, details of eczema severity and treatment, age at onset of eczema;
4. Comparators: definition of unexposed subjects, number of comparators;
5. Outcomes: definition and identification of cardiovascular outcomes (angina, myocardial infarction, coronary revascularisation, heart failure, cardiac arrhythmias, stroke and cardiovascular death), number of subjects with the outcome;

The definition of eczema that the authors state in the original study will be recorded in detail, and given due consideration in the assessment of study quality. We predict that the definition of eczema severity will be heterogeneous between studies. For example, severe eczema may be defined by the need for systemic treatment; however, this may be a source of misclassification bias. Therapeutic agents such as systemic corticosteroids may be used for alternative clinical indications and treatment is an imperfect proxy for defining severity. This uncertainty will be dealt with by including a description of how authors reported their eczema severity definitions in the data extraction stage, by paying particular attention to definitions of severity in the appraisal of quality, and in the discussion of the review. Where available, unadjusted and fully adjusted effect estimates will be recorded, along with details of confounders. Discrepancies will be resolved by discussion, with a third reviewer if necessary.

Outcomes

The primary outcome is the association between eczema and cardiovascular outcomes including angina, myocardial infarction, coronary revascularisation, heart failure, cardiac arrhythmias, stroke (all subtypes), and cardiovascular death. Where possible, effect estimates will include odds ratios, hazard ratios, and incident rate ratios, for cardiovascular outcomes in people with eczema compared to those without. Secondary outcomes include variation in the strength of association between severity of eczema and cardiovascular disease, whether the association varies by country, is altered by the treatment of eczema, by different CVD endpoints, or modified by age and gender.

For peer review only - http://bmjopen.bmj.com/site/about/guidelines.xhtml

Quality assessment

Critical appraisal will be independently recorded by reviewers to allow comparison by study quality. Risk of bias will be assessed by considering relevant domains to observational studies, including participant selection, measurement of variables, and controlling for confounding, in line with the Cochrane Collaboration's GRADE tool for assessing risk of bias, and the Newcastle-Ottawa Scale, in order to maximise relevance to non-randomised studies. Each domain will be rated with 'high', 'low' or 'unclear' with regards to the risk of bias, with free text explanations. Full results of this quality assessment will be included in the resulting review, with discussion of quality assessment in the narrative data synthesis.

Data synthesis

We will group studies by the cardiovascular outcome under study, and synthesise them narratively. No subgroup analyses are planned. We will only consider information on the interaction between eczema and covariates if this has been formally assessed in the original publication. We will perform meta-analyses on the association between eczema and specific cardiovascular outcomes with the help of a statistician to obtain a pooled effect estimate. We will assess statistical heterogeneity using the I^2 statistic. The pooled relative risk and its 95% confidence interval will be calculated using random effects models. If substantial heterogeneity is observed, we will explore the reasons for the heterogeneity in sensitivity analyses. Study characteristics and the effect estimates for the association between eczema and cardiovascular disease will be presented in full, in tabular form. Studies at high risk of bias will not be contained in the synthesis. We will look for publication bias using standard approaches including funnel plots and Egger tests. Preferred Reporting Items for Systematic Review and Meta-Analysis (PRISMA) guidelines[19] will be used to report the results of this study.

ETHICS AND DISSEMINATION

For peer review only - http://bmjopen.bmj.com/site/about/guidelines.xhtml

This systematic review protocol was registered with the International Prospective Register of Systematic Reviews (PROSPERO) on the 22nd of April 2016. Any amendments to the protocol will be documented on the PROSPERO site contemporaneously, with full explanation of any change.

Ethical approval is not required for this study as it is a systematic review. The results will be submitted for peer-reviewed publication, and for national and international presentation.

Authors' contributions:

AA contributed to the design of the study, was the guarantor, developed the search strategy and the PROSPERO protocol, and drafted the manuscript. AY contributed to the design of the study, approved the search strategy, and provided critical feedback on the PROSPERO protocol, and the final manuscript. MS approved the search strategy, and provided critical feedback on the PROSPERO protocol and the final manuscript. KA approved the search strategy, and provided critical feedback on the PROSPERO protocol and the final manuscript. LS approved the search strategy, and provided critical feedback on the PROSPERO protocol and the final manuscript. SML contributed to the conception and design of the study, and provided critical feedback on the search strategy, PROSPERO protocol and the final manuscript.

Funding statement: Publication of this manuscript was funded by a Wellcome Senior Clinical Fellowship to SML (205039/Z/16/Z). AA was supported by a small grant from the British Association of Dermatologists. LS is funded by a Wellcome Trust senior fellowship in clinical science. The Wellcome Trust and the British Association of Dermatologists played no role in the development of this study or the protocol.

Competing interests statement: All authors have completed the ICMJE uniform disclosure form at www.icmje.org/coi_disclosure.pdf and declare: AA reports grants from the British Association of Dermatologists during the conduct of the study. LS reports grants from Wellcome Trust during the conduct of the study; grants from Wellcome Trust, Medical Research Council, National Institute for

For peer review only - http://bmjopen.bmj.com/site/about/guidelines.xhtml

Health Research and the European Union outside the submitted work, personal fees from GSK for advisory work unrelated to the submitted work, grant funding from GSK for academic research unrelated to the submitted work, acts as an unpaid steering committee chair for AstraZeneca for a randomised trial unrelated to the submitted work, and is also a trustee of the British Heart Foundation. SML reports grants from Wellcome Senior Clinical fellowship in Science (205039/Z/16/Z) during the conduct of the study.

REFERENCES

1. Ronmark EP, Ekerljung L, Lotvall J, et al. Eczema among adults: prevalence, risk factors and relation to airway diseases. Results from a large-scale population survey in Sweden. *Br J Dermatol* 2012;166(6):1301-8. doi: 10.1111/j.1365-2133.2012.10904.x

2. Silverberg JI, Hanifin JM. Adult eczema prevalence and associations with asthma and other health and demographic factors: a US population-based study. *J Allergy Clin Immunol* 2013;132(5):1132-8. doi: 10.1016/j.jaci.2013.08.031

3. Williams H, Stewart A, von Mutius E, et al. Is eczema really on the increase worldwide? *J Allergy Clin Immunol* 2008;121(4):947-54 e15. doi: 10.1016/j.jaci.2007.11.004

4. Hansson GK. Inflammation, atherosclerosis, and coronary artery disease. *N Engl J Med* 2005;352(16):1685-95. doi: 10.1056/NEJMra043430

5. Hsue PY, Deeks SG, Hunt PW. Immunologic basis of cardiovascular disease in HIV-infected adults. *J Infect Dis* 2012;205 Suppl 3:S375-82. doi: 10.1093/infdis/jis200

6. Roifman I, Beck PL, Anderson TJ, et al. Chronic inflammatory diseases and cardiovascular risk: a systematic review. *Can J Cardiol* 2011;27(2):174-82. doi: 10.1016/j.cjca.2010.12.040

7. Mehta NN, Azfar RS, Shin DB, et al. Patients with severe psoriasis are at increased risk of cardiovascular mortality: cohort study using the General Practice Research Database. *Eur Heart J* 2010;31(8):1000-06. doi: 10.1093/eurheartj/ehp567

8. Tamagawa-Mineoka R, Katoh N, Ueda E, et al. Platelet-derived microparticles and soluble P-selectin as platelet activation markers in patients with atopic dermatitis. *Clin Immunol* 2009;131(3):495-500. doi: 10.1016/j.clim.2009.01.006

9. Tamagawa-Mineoka R, Katoh N, Ueda E, et al. Elevated platelet activation in patients with atopic dermatitis and psoriasis: increased plasma levels of beta-thromboglobulin and platelet factor 4. *Allergol Int* 2008;57(4):391-6. doi: 10.2332/allergolint.O-08-537

10. Hjuler KF, Bottcher M, Vestergaard C, et al. Increased Prevalence of Coronary Artery Disease in Severe Psoriasis and Severe Atopic Dermatitis. *Am J Med* 2015;128(12):1325-34 e2. doi: 10.1016/j.amjmed.2015.05.041

11. Roubille C, Richer V, Starnino T, et al. The effects of tumour necrosis factor inhibitors, methotrexate, non-steroidal anti-inflammatory drugs and corticosteroids on cardiovascular events in rheumatoid arthritis, psoriasis and psoriatic arthritis: a systematic review and meta-analysis. *Ann Rheum Dis* 2015;74(3):480-9. doi: 10.1136/annrheumdis-2014-206624

12. Wei L, MacDonald TM, Walker BR. Taking glucocorticoids by prescription is associated with subsequent cardiovascular disease. *Ann Intern Med* 2004;141(10):764-70.

13. Standl M, Tesch F, Baurecht H, et al. Association of Atopic Dermatitis with Cardiovascular Risk Factors and Diseases. *J Invest Dermatol* 2017;137(5):1074-81. doi: 10.1016/j.jid.2016.11.031

For peer review only - http://bmjopen.bmj.com/site/about/guidelines.xhtml

14. Su VY, Chen TJ, Yeh CM, et al. Atopic dermatitis and risk of ischemic stroke: a nationwide population-based study. *Ann Med* 2014;46(2):84-9. doi: 10.3109/07853890.2013.870018
15. Riis JL, Vestergaard C, Hjuler KF, et al. Hospital-diagnosed atopic dermatitis and long-term risk of myocardial infarction: a population-based follow-up study. *BMJ Open* 2016;6(11):e011870. doi: 10.1136/bmjopen-2016-011870
16. Silverberg JI, Greenland P. Eczema and cardiovascular risk factors in 2 US adult population studies. *J Allergy Clin Immunol* 2015;135(3):721-8 e6. doi: 10.1016/j.jaci.2014.11.023
17. Zhang A, Silverberg JI. Association of atopic dermatitis with being overweight and obese: a systematic review and metaanalysis. *J Am Acad Dermatol* 2015;72(4):606-16 e4. doi: 10.1016/j.jaad.2014.12.013
18. Cardwell CR, Shields MD, Carson DJ, et al. A meta-analysis of the association between childhood type 1 diabetes and atopic disease. *Diabetes Care* 2003;26(9):2568-74.
19. Moher D, Liberati A, Tetzlaff J, et al. Preferred reporting items for systematic reviews and meta-analyses: the PRISMA statement. *BMJ* 2009;339:b2535. doi: 10.1136/bmj.b2535

For peer review only

For peer review only - http://bmjopen.bmj.com/site/about/guidelines.xhtml

Supplementary Appendix 1

Ovid MEDLINE Search Strategy

1. Dermatitis, Atopic/

2. exp Eczema/

3. (eczem* or atopic dermatit*).mp. [mp=title, abstract, original title, name of substance word, subject heading word, keyword heading word, protocol supplementary concept word, rare disease supplementary concept word, unique identifier, synonyms]

4. or/1-3

5. intracranial embolism/

6. exp Intracranial Hemorrhages/

7. exp Intracranial Arterial Diseases/

8. exp Brain Ischemia/

9. (intracranial embolism* or intracranial h?emorrhage* or intracranial arterial disease* or intracranial thrombos* or stroke* or cerebrovascular accident* or cerebrovascular diseas* or cva or cerebral artery diseas* or brain isch* or brain infarct* or brain h?emorrhag* or occlusive cerebrovascular disease*).mp. [mp=title, abstract, original title, name of substance word, subject heading word, keyword heading word, protocol supplementary concept word, rare disease supplementary concept word, unique identifier, synonyms]

10. exp Cardiovascular Diseases/

11. (myocardial isch?emia* or heart arrest* or heart attack* or myocardial infarct* or acute coronary syndrome* or angina* or isch?emic heart diseas* or coronary arter* or heart fail* or congestive cardiac fail* or ccf or lvf or left ventricular fail* or rvf or right ventricular fail* or heart right ventricle fail* or heart left ventricle fail* or af or cardiovascular diseas* or heart diseas* or vascular diseas* or arrhythmia* or abnormal heart rhythm* or atrial flutter* or heart block* or svt* or supraventricular tachycardia* or bundle branch block*).mp. [mp=title, abstract, original title, name of substance word, subject heading word, keyword heading word, protocol supplementary concept word, rare disease supplementary concept word, unique identifier, synonyms]

12. exp Myocardial Revascularization/

13. exp Percutaneous Coronary Intervention/

14. (myocardial revasculari?ation* or percutaneous coronary intervention* or heart muscle revasculari?ation* or coronary revasculari?ation* or percutaneous transluminal angioplast* or coronary artery obstruction* or transluminal coronary angioplast* or coronary artery surger* or interventional cardiovascular procedure*).mp. [mp=title, abstract, original title, name of substance word, subject heading word, keyword heading word, protocol supplementary concept word, rare disease supplementary concept word, unique identifier, synonyms]

15. (cardiac arrest* or heart death* or card* death* or (cardiovascular adj3 mortalit*)).mp. [mp=title, abstract, original title, name of substance word, subject heading word, keyword heading word, protocol supplementary concept word, rare disease supplementary concept word, unique identifier, synonyms]

16. or/5-15

17. 4 and 16

For peer review only

The association between eczema and major cardiovascular outcomes in population based studies: a systematic review protocol

PRISMA-P (Preferred Reporting Items for Systematic review and Meta-Analysis Protocols) 2015 checklist: recommended items to address in a systematic review protocol

Section Topic	Item Number	Checklist Item	Corresponding page number
**Administrative Information**			
Title: Identification	1a	Identify the report as a protocol of a systematic review	1
Title: Update	1b	If the protocol is for an update of a previous systematic review, identify as such	N/A
Registration	2	If registered, provide the name of the registry (such as PROSPERO) and registration number	2
Authors: Contact	3a	Provide name, institutional affiliation, e-mail address of all protocol authors; provide physical mailing address of corresponding author	1
Authors: Contributions	3b	Describe contributions of protocol authors and identify the guarantor of the review	9
Amendments	4	If the protocol represents an amendment of a previously completed or published protocol, identify as such and list changes; otherwise, state plan for documenting important protocol amendments	9
Support: Sources	5a	Indicate sources of financial or other support for the review	9
Support: Sponsor	5b	Provide name for the review funder and/or sponsor	9
Support: Role of sponsor or funder	5c	Describe roles of funder(s), sponsor(s), and/or institution(s), if any, in developing the protocol	9
**Introduction**			
Rationale	6	Describe the rationale for the review in the context of what is already known	4
Objectives	7	Provide an explicit statement of the question(s) the review will address with reference to participants, interventions, comparators, and outcomes (PICO)	4, 5
**Methods**			
Eligibility criteria	8	Specify the study characteristics (such as PICO, study design, setting, time frame) and report characteristics (such as years considered, language, publication status) to be used as criteria for eligibility for the review	5
Information sources	9	Describe all intended information sources (such as electronic databases, contact with study authors, trial registers or other grey literature sources) with planned dates of coverage	6
Search strategy	10	Present draft of search strategy to be used for at least one electronic database, including planned limits, such that it could be repeated	Provided as a separate upload
Study records: Data management	11a	Describe the mechanism(s) that will be used to manage records and data throughout the review	6

For peer review only

Study records: Selection process	11b	State the process that will be used for selecting studies (such as two independent reviewers) through each phase of the review (that is, screening, eligibility and inclusion in meta-analysis)	6
Study records: Data collection process	11c	Describe planned method of extracting data from reports (such as piloting forms, done independently, in duplicate), any processes for obtaining and confirming data from investigators	6
Data items	12	List and define all variables for which data will be sought (such as PICO items, funding sources), any pre-planned data assumptions and simplifications	6, 7
Outcomes and prioritisation	13	List and define all outcomes for which data will be sought, including prioritization of main and additional outcomes, with rationale	7
Risk of bias in individual studies	14	Describe anticipated methods for assessing risk of bias of individual studies, including whether this will be done at the outcome or study level, or both; state how this information will be used in data synthesis	8
Data synthesis	15a	Describe criteria under which study data will be quantitatively synthesised	8
	15b	If data are appropriate for quantitative synthesis, describe planned summary measures, methods of handling data and methods of combining data from studies, including any planned exploration of consistency (such as I2, Kendall's τ)	8
	15c	Describe any proposed additional analyses (such as sensitivity or subgroup analyses, meta-regression)	8
	15d	If quantitative synthesis is not appropriate, describe the type of summary planned	8
Meta-bias(es)	16	Specify any planned assessment of meta-bias(es) (such as publication bias across studies, selective reporting within studies)	8
Confidence in cumulative evidence	17	Describe how the strength of the body of evidence will be assessed (such as GRADE)	8

From: Shamseer L, Moher D, Clarke M, Ghersi D, Liberati A, Petticrew M, Shekelle P, Stewart L, PRISMA-P Group. Preferred reporting items for systematic review and meta-analysis protocols (PRISMA-P) 2015: elaboration and explanation. BMJ. 2015 Jan 2;349(jan02 1):g7647.

For peer review only
